# Health, Psychological Distress, and Functioning During the COVID-19 Pandemic Among Danish Adults with and Without a Preexisting Mental Illness

**DOI:** 10.3390/ijerph22081260

**Published:** 2025-08-12

**Authors:** Per Vendsborg, Nanna Schneekloth Jarlstrup, Sofie H. Hoffmann, Merete Nordentoft, Christoph U. Correll, Marco Solmi, Trevor Thompson, Andrés Estradé, Trine Toft Sørensen, Lau Caspar Thygesen

**Affiliations:** 1 Danish Mental Health Fund (Psykiatrifonden), Hejrevej 43, DK-2400 Copenhagen, Denmark; 2 Centre for Childhood Health, Islands Brygge 41, DK-2300 Copenhagen, Denmark; nsj@cslt.dk; 3 National Institute of Public Health, University of Southern Denmark, Studiestræde 6, DK-1455 Copenhagen, Denmark; sohh@sdu.dk (S.H.H.); trins@sdu.dk (T.T.S.); lct@sdu.dk (L.C.T.); 4 CORE—Copenhagen Research Centre for Mental Health, Mental Health Centre Copenhagen, Copenhagen University Hospital, DK-2900 Copenhagen, Denmark; mn@dadlnet.dk; 5 Department of Clinical Medicine, Faculty of Health and Medical Sciences, University of Copenhagen, DK-1165 Copenhagen, Denmark; 6 The Lundbeck Foundation Initiative for Integrative Psychiatric Research (iPSYCH), DK-8210 Aarhus, Denmark; 7 German Center for Mental Health (DZPG), Partner Site Berlin, 10117 Berlin, Germany; ccorrell@northwell.edu; 8 Donald and Barbara Zucker School of Medicine at Hofstra/Northwell, New York, NY 11549, USA; 9 Department of Child and Adolescent Psychiatry, Charité Universitätsmedizin Berlin, 10117 Berlin, Germany; marco.solmi83@gmail.com; 10 Department of Mental Health, The Ottawa Hospital, Ottawa, ON K1Y 4E9, Canada; 11 Ottawa Hospital Research Institute (OHRI) Clinical Epidemiology Program, University of Ottawa, Ottawa, ON K1H 8L6, Canada; 12 Department of Psychiatry, University of Ottawa, Ottawa, ON K1N 6N5, Canada; 13 Centre for Chronic Illness and Aging, University of Greenwich, London SE10 9LS, UK; t.thompson@greenwich.ac.uk; 14 Early Psychosis: Interventions and Clinical-Detection (EPIC) Lab, Department of Psychosis Studies, Institute of Psychiatry, Psychology& Neuroscience, King’s College London, London WC2R 2LS, UK; andres.estrade_vaz@kcl.ac.uk

**Keywords:** mental health, mental illness, COVID-19, corona, pandemic, lockdown, distress, function

## Abstract

The aim of this paper was to evaluate health, psychological distress, and functioning during the COVID-19 pandemic among Danish adults with and without a history of mental illness. Data were drawn from three online surveys conducted in May 2020 (n = 3134), January 2021 (n = 1170), and January 2022 (n = 1174) as part of the Danish contribution to the Collaborative Outcomes study on Health and Functioning during Infection Times (COH-FIT). The prevalence of mental and physical health issues, psychological distress (stress, sleep problems, loneliness, and boredom) and levels of functioning (self-care, interpersonal relationships, hobbies/leisure, and work/education) were evaluated at four different time points stratified by history of mental illness. Findings indicated that physical health was not differentially affected between people with and without prior mental illness. However, mental health declined significantly more among respondents with a history of mental illness. While levels of stress did not differ between the two groups, boredom was more pronounced in May 2020 among those with prior mental illness. Loneliness was significantly higher in this group in January 2021. Sleep disturbances were more pronounced for respondents with former mental illness during the whole period. A decline in functioning was observed in people both with and without a former mental illness. It seemed a little more pronounced for people with mental illness but seldom reached statistical significance. For all measures of health, distress, and functioning, 10–20% of respondents reported improvements in health, distress, and functioning during the pandemic, with stress showing the most improvement—one third of participants reported feeling less stressed. In most of the parameters measured, the influence of the COVID-19 pandemic seemed to decrease with time. However, the effects were not uniform, and more investigations are needed to understand the whole picture.

## 1. Introduction

The Collaborative Outcomes study on Health and Functioning during Infection Times (COH-FIT) [1] is an international survey study conducted in many countries and translated into 30 languages. We report data from the Danish part of the study.

In Denmark, the government began implementing measures to control the transmission of the virus in March 2020, including the lockdown of institutions, workplaces, and cultural and leisure activities. Public gatherings were also restricted. These measures were gradually eased during the summer and fall of 2020 but were reintroduced and intensified during the winter of 2020–2021 due to rising infection rates. Eventually, the lockdowns and restrictions came to an end.

The lockdowns and restrictions were expected to have not only social and economic consequences but also mental health impacts, particularly related to fear of infection and social isolation. These consequences placed stress on the population and influenced mental health. The pandemic affected mental health of both the general population and individuals with pre-existing mental illnesses. Later reviews comprising more publications have shown that the effects of the pandemic are not straightforward and homogenous between populations [2].

The Global Burden of Disease (GBD) study analyzed global data and found that the COVID-19 pandemic has had a negative impact on mental health worldwide. Rates of anxiety and depression increased substantially, especially in areas with the highest infection rates, with females and younger individuals being more affected [3]. A similar deterioration in mental health has also been reported in several other reviews [4,5,6]. However, previous reviews have yielded mixed findings regarding the impact of the pandemic on individuals with pre-existing mental illness. For example, some studies reported a more severe worsening in this group [6,7], suggesting greater vulnerability. In contrast, other reviews concluded that the general population experienced only mild or no mental health deterioration [2,8], and in some cases, individuals with mental illness were found to be equally or even less affected [2,9]. These discrepancies may be due to contextual differences, variations in measurement methods, or even potential protective factors such as reduced daily stress during lockdowns. A more systematic comparison of findings is needed to better understand the conditions under which mental illness serves as a risk or resilience factor during pandemics.

One explanation could be that people with different mental illnesses exhibited different vulnerabilities to the pandemic. It has been shown that patients with serious mental illness may have been less affected by the pandemic [10,11], possibly due to a floor effect or because social isolation reduced interpersonal stress. Another explanation could be that different age groups had varying susceptibility [7,12]. A review by Hossein et al., 2020 reported that most studies found younger people to be more vulnerable than older ones to the adverse effects of the pandemic, although a single study observed the opposite [6]. Similarly, in the global COH-FIT sample, those at younger age were also more vulnerable to experience greater worsening of wellbeing, psychopathology [13,14], and suicidality [15].

Furthermore, the hope for a forthcoming vaccine and the final receipt of protective vaccination may have played a mitigating role [16]. Additionally, mental health can also be adversely impacted by the effect of the virus and infection itself, which can have long-term negative consequences in a vulnerable subgroup of individuals. [17]

In this study, we examine how physical and mental health, stress, sleep, loneliness, boredom, and functioning—measured by self-care, interpersonal relationships, hobbies/leisure activities, and work/education—varied in the Danish population with and without mental illness during the first two years of the COVID-19 pandemic

## 2. Methods

### 2.1. Setting

The first COVID-19 case in Denmark was registered on the 26 February 2020. The rapid spread throughout Denmark resulted in a national lockdown on the 11 March 2020, which included several societal restrictions such as assembly bans, social distancing, and closing of educational institutions, daycare facilities, and public workplaces. Restaurants, sport facilities, and cultural institutions were also closed. In May and June 2020, the number of hospitalizations decreased, followed by the termination of most restrictions. When hospitalizations increased during fall and winter 2020/2021, most restrictions were reintroduced. Through 2021, the restrictions were downscaled simultaneously with a decreasing number of hospitalizations during winter 2021/2022. On the 1 February 2022, all restrictions were removed, and COVID-19 was no longer defined as a critical societal disease.

### 2.2. Data

The Collaborative Outcomes study on Health and Functioning during Infection Times (COH-FIT) is a large-scale survey, including more than 50 countries from all six inhabited continents. The COH-FIT aims to identify risk factors affecting the general population and vulnerable subgroups during the COVID-19 pandemic. Additional information about this global study is available elsewhere [1]. In this study, we present results from the Danish part of the COH-FIT. Data was collected through online questionnaires in separate samples in May 2020, January 2021, and January 2022, a period in which COVID-19 hospitalizations and societal restrictions mandated by the government varied greatly in Denmark. In May 2020, the questionnaire was promoted by the Danish Mental Health Fund and the National Institute of Public Health, University of Southern Denmark, in newsletters sent to members and in news media. To retrieve representative samples of Danish adults (18+ years) according to sex, age, geographic location, educational level, and occupation, two subsequent data collections in January 2021 (11 to the 20 January 2021) and in January 2022 (14 to the 20 January 2022) were performed by a survey agency using an already established panel data set.

### 2.3. Measures

Physical and mental health, distress (stress, sleep problems, loneliness, and boredom), and functioning levels were assessed at each time point by a self-report. Respondents were asked to recall and rate on a scale from 0 to 100 their health, distress, and functioning during the actual past two weeks and in the last two weeks before the COVID-19 pandemic outbreak. For each respondent, the values were compared and registered as better, worse, or unchanged. The reported results are the percentage of respondents reporting worse, unchanged, or better for each parameter at the three time points the measurements took place. The validity of scales used for measuring the parameters has been confirmed [18].

The respondents were asked about their actual condition, followed by a question about their condition two weeks prior to the COVID-19 pandemic. Rather than recalling specific past states, they only needed to assess whether their current condition was better, worse, or unchanged compared to the pre-pandemic period. That could cause a recall bias, but they did not have to recall their condition before. They only had to think: Am I better, worse, or unchanged today during the epidemic than before? That made it easier. If unchanged they rated the same value as an actual condition, and there was no need to have an interval for unchanged. This simplification lessened the risk of recall bias.

To obtain information about mental illness, respondents were asked whether they had ever been diagnosed with mental health conditions by a doctor or psychologist. Respondents indicating at least one mental health condition were defined as having a mental illness, while respondents reporting no diagnoses were defined as having no mental illness.

### 2.4. Weight

To achieve three comparable samples reflecting populations with the same distribution of sex, age, educational level, and occupation, the data collected in May 2020 was weighted in accordance with the representative sample from January 2021. Respondents with missing information on educational level in the samples collected in May 2020 (n = 10) and January 2022 (n = 1) were categorized as having a college/university/PhD, which constituted the largest group.

### 2.5. Statistical Analysis

Descriptive statistics were used to evaluate the prevalence of mental and physical health, distress, and functioning levels at four different time points: before the COVID-19 pandemic, in May 2020, in January 2021, and in January 2022. All analyses were stratified by pre-existing mental illness. Differences in prevalences were tested with chi-squared (χ^2^) tests. All analyses were conducted in STATA, version 17.0.

## 3. Results

In May 2020, a total of 3134 individuals responded to the questionnaire, while, respectively, 1170 and 1174 responded in January 2021 and January 2022 (see Table 1). In the unweighted sample from May 2020, the proportion of women (84%), respondents between the age of 50–59 years (26%) and those with college/university/PhD educational level (86%) were overrepresented compared to the representative sample from January 2021 and January 2022 seen in the first and second column (see Table 1). Further, a higher proportion were without an occupation (40%) in May 2020 compared to the sample in January 2021 (22%) and January 2022 (17%) and had at least one mental illness (45%) (15% in January 2021 and 17% in January 2022).

**Figure 1:**
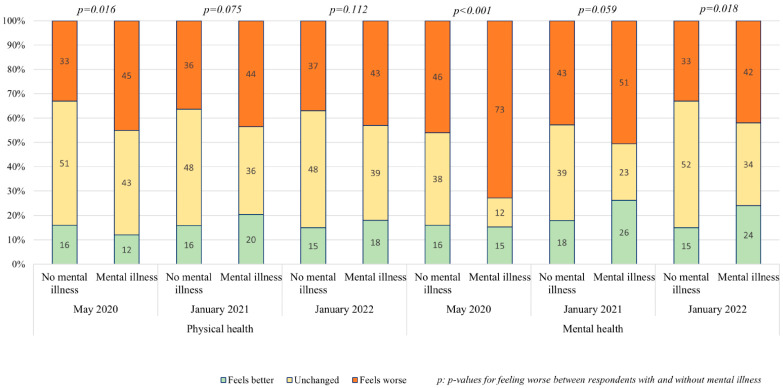
Self-reported change in physical and mental health associated with COVID-19 in May 2020, January 2021, and January 2022 among individuals with and without mental illness. The 6 first columns show physical health, and the 6 last represent mental health across the 3 time periods.

Among the respondents with no mental illness, about one third reported a worsening of their physical health over time. For the respondents with mental illness, the worsening was experienced for about half of them, also constant over time. The difference between respondents without and with mental illness was not statistically significant. Mental health worsened for all the respondents, but the deterioration was significantly more pronounced among respondents with mental illness. Furthermore, this group reported a lower level of mental health prior to the pandemic, further increasing the gap in mental health. Over time, the decline in mental health lessened for both groups, with individuals with prior mental disorder showing greater recovery. Results are presented in Figure 1.

**Figure 2:**
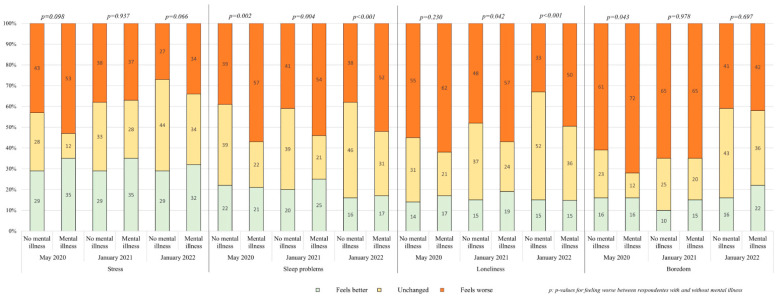
Self-reported change in symptoms of psychological distress associated with COVID-19 in May 2020, January 2021, and January 2022 among individuals with and without mental illness. Stress, sleep problems, loneliness, and boredom are shown in pairs of 6 columns each with and without former mental illness across the 3 time periods.

About half of the respondents reported experiencing worse stress in May 2020. The worsening decreased with time to one third in January 2022. There was no difference between respondents without and with mental illness. Boredom was a major problem in May, affecting approximately three-quarters of respondents with mental illness and two-thirds of those without—a significant difference. Although boredom decreased over time, it remained a major issue, with no significant difference between the two groups. Loneliness was reported as worse in May by about half of the respondents without mental illness and two thirds of those with mental illness, though the difference not initially significant. It decreased with time for all respondents but less so for the respondents with mental illness, leading to a significant difference in the second and third sampling periods. Sleeping problems were reported as worse by about one-third of respondents without mental illness and about half of those with mental illness, with the difference remaining significant at all three time points. Results are presented in Figure 2.

**Figure 3:**
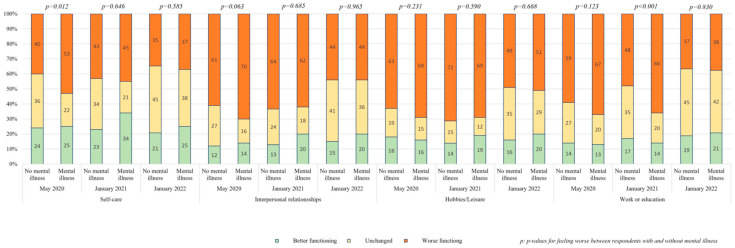
Self-reported change in functioning associated with COVID-19 in May 2020, January 2021, and January 2022 among individuals with and without mental illness. Self-care, interpersonal relationships, hobbies/leisure, and work/education are depicted with each 6 columns pairwise with and without former mental illness across the 3 time periods.

Functioning levels were estimated by changes in self-care, interpersonal relationships, hobbies/leisure, and work/education. Of the four parameters, self-care was least compromised, with about half of respondents reporting worsening and minimal differences between those with and without mental illness. Only the sampling in May showed a significant difference. Interpersonal relationships and hobbies/leisure showed worsening in approximately half to two-thirds of respondents but no difference between those with and without mental illness. Work/education similarly showed deterioration in about half to two-thirds of respondents, with little difference between the respondents with and without mental illness. Only the middle sampling in January 2021 showed an increased number of respondents with mental illness that reached significance. Results are presented in Figure 3.

## 4. Discussion

During the COVID-19 pandemic, we assessed changes in mental and physical health, psychological distress, and functioning levels over a two-year period in people with and without former mental illness.

Mental health decreased significantly more among respondents with a history of mental illness. Boredom was more pronounced in May 2020 in people with former mental illness, and loneliness was significantly higher in January 2021. Sleep disturbances were more pronounced for respondents with former mental illness throughout the entire period.

Across all parameters and respondent groups, 10–20% reported feeling better during the pandemic. This was especially notable for stress, where one third of respondents felt an improvement.

That respondents without former mental illness felt negatively affected by the pandemic is in accordance with most reviews, although some report only minimal effects [2,3,4,5,6,8].

Greater worsening of mental health, compared to people without former mental health, in a population with mental illnesses is consistent with some reviews [7], but not with others [2,9]. The latter reviews present mixed results and often even report positive effects from the pandemic. These results have been explained by various theories, e.g., the actual daily work stress experienced by people with mental illness was lessened because they could stay home and maybe some did not feel so isolated or lonely because families had to be more together.

Increase in psychological distress was seen especially in the form of sleeping problems and partly in loneliness, with a more pronounced reaction for respondents with former mental illness, as also found by others [5,6]. Sleep disturbances were consistently more prevalent among respondents with a history of mental illness across all time points, with 57% in May 2020, 54% in January 2021, and 52% in January 2022, compared to 39%, 41%, and 38% among those without mental illness, respectively. This persistent difference suggests a chronic vulnerability in this subgroup, potentially exacerbated by pandemic-related disruptions to routine and treatment access.

Contrary to the findings of Ahmed et al., 2023, who reported significantly greater functional decline among individuals with pre-existing mental illness [2], our study observed no consistent differences between groups across domains such as interpersonal relationships, self-care, and occupational functioning. This discrepancy may stem from methodological differences, including the reliance on self-reported function, differences in timing, or cultural/contextual variation in the Danish sample that may have buffered functional impact. In Denmark, Pedersen et al., 2022 reported poorer mental health during lockdowns, particularly among people with former mental illness [19]. Thygesen et al., 2021 found that there was a decline in mental wellbeing during the pandemic [20], but it was small and most pronounced for people without former mental illness compared to people with former illness.

Our study has several strengths. The rapid collection of data in May 2020 allowed us to assess the physical and mental health of a large number of Danes at a critical point in time. Combined with the two other survey waves, we were able to assess the influence on physical and mental health during the pandemic. Furthermore, the study population included a high number of individuals with preexisting mental illness (45%), which improved statistical power in the stratified analyses among individuals with and without preexisting illness. Lastly, by recruiting a representative sample of Danish adults in January 2021 and January 2022, the data collected in May 2020 could be weighted to reflect the broader adult population in Denmark.

Some limitations also need to be acknowledged. The May 2020 survey may have been subject to self-selection bias, whereby individuals with a particular interest in the study topic were more likely to participate. Recall bias may have influenced the results of physical and mental health prior to the pandemic, especially for the data collected in January 2021 and January 2022. This risk was mitigated to some extent by the simplicity of the retrospective question—respondents were only asked whether their current condition was better, worse, or unchanged. Prospective cohorts that were investigated some time before and then during the pandemic could alleviate this problem. The study by Thygesen et al., 2021 is an example [20]. Nevertheless, when examining the potential for recall bias in the COH-FIT study with respect to the WHO-5 and P-factor scores, we found neither an upward nor downward drift at a population level in pre-pandemic assessments throughout the interpandemic recall period [13]. Furthermore, the categorization of mental illness in our study was rather simple. Arguably, employing a more detailed diagnostic framework—encompassing a wider range of mental health conditions—might have provided more nuanced findings. A review with that scope could maybe be produced now taking into consideration the large number of papers produced since the epidemic.

## 5. Conclusions

The COVID-19 pandemic had a predominantly negative impact on physical and mental health, psychological distress, and overall functioning in the general population. This effect was particularly pronounced in individuals with a history of mental illness, specifically in the domains of mental health and sleep. Our findings agree with the varying results reported in the existing literature.

## Figures and Tables

**Table 1:** Characteristics of the study population by time period.

	May 2020 ^a^ (n = 3134) n (%)	May 2020 ^b^ (n = 3078) n (%)	January 2021 (n = 1170) n (%)	January 2022 (n = 1174) n (%)
Sociodemographic characteristics				
Sex				
Men	496 (16)	1445 (47)	560 (48)	526 (45)
Women	2638 (84)	1634 (53)	610 (52)	648 (55)
Age				
18–29 years	384 (12)	874 (28)	333 (28)	320 (27)
30–39 years	465 (15)	417 (14)	180 (15)	191 (16)
40–49 years	677 (22)	548 (18)	204 (17)	197 (17)
50–59 years	805 (26)	530 (17)	199 (17)	217 (18)
60–69 years	590 (19)	556 (18)	202 (17)	203 (17)
70+ years	213 (7)	153 (5)	52 (4)	46 (4)
Mean age (min; max)	49 (18;84)	44 (18;84)	43 (18;76)	43 (18;86)
Educational level				
None/primary education	217 (7)	874 (28)	361 (31)	216 (18)
High school/vocational school	230 (7)	1079 (35)	407 (35)	327 (28)
College/university degree/PhD	2687 (86)	1126 (37)	402 (34)	631 (54)
Occupation				
Not working	1243 (40)	711 (23)	263 (22)	194 (17)
Not working in health care	1303 (41)	2081 (68)	802 (69)	837 (71)
Working in health care	574 (18)	258 (8)	104 (9)	142 (12)
Missing	14 (1)	28 (1)	1 (0)	1 (0)
Health and wellbeing				
Mental illness				
No mental illness	1629 (52)	1480 (48)	956 (82)	946 (80)
At least one mental illness	1322 (42)	1344 (44)	172 (15)	194 (17)
Missing	183 (6)	254 (8)	42 (3)	34 (3)

Note: ^a^ unweighted, ^b^ weighted on sex, age, educational level, and occupation. Due to rounding of weights, the sum of the cells may not equal the total exactly.

## Data Availability

Data can be found by writing to the authors.
